# Phytochrome B photobodies are comprised of phytochrome B and its primary and secondary interacting proteins

**DOI:** 10.1038/s41467-023-37421-z

**Published:** 2023-03-27

**Authors:** Chanhee Kim, Yongmin Kwon, Jaehoon Jeong, Minji Kang, Ga Seul Lee, Jeong Hee Moon, Hyo-Jun Lee, Youn-Il Park, Giltsu Choi

**Affiliations:** 1 Department of Biological Sciences, Korea Advanced Institute of Science and Technology, Daejeon, 34141 Korea; 2 Disease Target Structure Research Center, Korea Research Institute of Bioscience and Biotechnology, Daejeon, 34141 Korea; 3 College of Pharmacy, Chungbuk National University, Cheongju, Chungbuk 28160 Korea; 4 Plant Systems Engineering Research Center, Korea Research Institute of Bioscience and Biotechnology, Daejeon, 34141 Korea; 5 Department of Biological Sciences, Chungnam National University, Daejeon, 34134 Korea

**Keywords:** Light responses, Plant molecular biology, Proteomics

## Abstract

Phytochrome B (phyB) is a plant photoreceptor that forms a membraneless organelle called a photobody. However, its constituents are not fully known. Here, we isolated phyB photobodies from *Arabidopsis* leaves using fluorescence-activated particle sorting and analyzed their components. We found that a photobody comprises ~1,500 phyB dimers along with other proteins that could be classified into two groups: The first includes proteins that directly interact with phyB and localize to the photobody when expressed in protoplasts, while the second includes proteins that interact with the first group proteins and require co-expression of a first-group protein to localize to the photobody. As an example of the second group, TOPLESS interacts with PHOTOPERIODIC CONTROL OF HYPOCOTYL 1 (PCH1) and localizes to the photobody when co-expressed with PCH1. Together, our results support that phyB photobodies include not only phyB and its primary interacting proteins but also its secondary interacting proteins.

## Abstract (web summary)

Phytochrome is a photoreceptor forming a membraneless organelle called a photobody. The authors isolated the photobody and found that the photobody is made of not only phytochrome but also its primary and secondary interacting proteins.

## Introduction

Phytochrome (phy, phyA to phyE in *Arabidopsis*) is a plant photoreceptor that detects red (670 nm) and far-red (730 nm) light through its covalently linked light absorbing chromophore called phytochromobilin^1,2^. Phytochromobilin undergoes reversible isomerization when irradiated with red and far-red light, leading to structural changes in the phy holoprotein^3^. The phy isomer induced by red light (Pfr) is biologically active and enters the nucleus, while the phy isomer induced by far-red light (Pr) is biologically inactive and exits the nucleus^4^. Pfr can also be thermally converted to Pr, allowing phy to sense both light and temperature^5,6^. Among the phys, phyA is the major phy that promotes light responses under deep shade, dark-to-light transition, and high-intensity light, while phyB is the major phy that promotes light responses under red-enriched light^2^. Phy promotes light responses by interacting with a set of proteins in the nucleus; these include PHYTOCHROME INTERACTING FACTORS (PIFs), bHLH transcription factors that promote shade responses^7^, and CONSTITUTIVELY PHOTOMORPHOGENIC 1 (COP1)/SUPPRESSOR OF PHYA-105 (SPA) complexes, which are ubiquitin E3 ligases that ubiquitinate factors such as HY5 to promote light responses^8^.

An interesting but less well-understood phenomenon in phy signaling is the formation of photobodies by liquid-liquid phase separation (LLPS) in the nucleus^9–11^. The sizes and numbers of photobodies within a nucleus are dynamic; they partly depend on the level of Pfr, as they undergo dynamic changes according to the R:FR ratio, light intensity, and temperature^5,12–14^. In addition to these external conditions, a few genetic factors have been identified to regulate phyB photobody formation by influencing the Pfr level. Among them, PCH1 and PCH1-like (PCHL) interact with phyB and stabilize the photobody by suppressing the thermal reversion of phyB from Pfr to Pr^15–17^, while the *hy1* mutation reduces the formation of the photobody by abolishing chromophore biosynthesis^13^. Mutations in a set of nuclear-encoded chloroplast genes (HMR, PAP8, RCB, NCP) encoding proteins that are dually localized in both the chloroplast and the nucleus also greatly reduce the sizes and numbers of photobodies^18–21^. Although the presence of photobodies is quite conspicuous in light-grown seedlings, their role is not fully understood^9,10^. Mutational analyses have indicated that some phyB mutant alleles fail to form photobodies but still promote light responses, albeit weakly^13,22^. This suggests that photobodies are not essential for phyB signaling, but rather enhance it. At the molecular level, the photobody has been proposed to be a site for transcription, protein sequestration, and protein degradation^9,10,23^.

A few phyB-interacting signaling components have been shown to be localized to the phyB photobody. PIFs and COP1/SPA complexes are two key phyB-interacting signaling components. The interaction of phyB leads PIFs to be phosphorylated by kinases such as PHOTEREGULATORY PROTEIN KINASES (PPKs)^24^, and the phosphorylated PIFs undergo polyubiquitination by ubiquitin E3 ligases, such as COLD TEMPERATURE-GERMINATING 10 (CTG10) for PIF1^25^, LIGHT-RESPONSE BRIC-A-BRACK/TRAMTRACK/BROAD (LRBs) for PIF3^26^, and BLADE-ON-PETIOLE (BOPs) for PIF4^27^. The ubiquitinated PIFs are then degraded by the 26 S proteasome^28^. Among these signaling components, PIF3 is transiently localized to the photobody when dark-grown transgenic seedlings expressing PIF3-GFP are transferred to red light, presumably due to the rapid degradation of PIF3 protein^29^ whereas the more stable PIF7 is localized to the photobody even after 4 h of white-light exposure in a long-day condition^30^. PPKs, which are also known as MUT9P-LIKE KINASES 1-4 (MLK1-MLK4), form nuclear bodies^24,31–33^, but it is not known if these PPK1 nuclear bodies are identical to the phyB photobodies. PhyB also interacts with and inhibits COP1/SPA complexes by excluding COP1 from the nucleus, degrading SPA proteins, or reorganizing COP1/SPA complexes^8^. Unlike the transient localization of PIF3 to the photobody, both COP1 and SPA1 proteins are more stably localized to the photobody^34–37^. In addition to the PIFs and COP1/SPA complexes, phyB also interacts with a few other proteins to regulate their activities. For example, phyB interacts with TANDEM ZINC-FINGER-PLUS3 (TZP) and enhances its targeting to the FT promoter to regulate flowering^23^. PhyB interacts with B-BOX CONTAINING PROTEIN 4 (BBX4) and COLD REGULATED 27/28 (COR27/28) and stabilizes them to regulate light responses either by inhibiting the transcriptional activation activity of PIF3 (BBX4) or inhibiting the expression of circadian clock genes (COR27/28)^38–40^. TZP and BBX4 form nuclear bodies that overlap with the phyB photobody^23,39^, while COR27 and COR28 do not form nuclear bodies^40^. Interestingly, the TZP nuclear body and the phyB photobody are dismantled by the RNA polymerase inhibitor, α-amanitin, suggesting that active transcription is required to maintain these nuclear bodies^23^. PhyB-interacting proteins that regulate alternative splicing (SFPS, RRC1, SMP2) have also been shown to localize to the phyB photobody^41–43^. Collectively, these previous reports show that phyB photobodies include some of phyB-interacting proteins. Thus, the systematic identification of photobody components would be useful to further investigate the role of phyB nuclear photobodies.

Here, we report the isolation of phyB photobodies and the identification of their components. We isolated phyB photobodies from transgenic *Arabidopsis* leaves expressing phyB-GFP using fluorescence-activated particle sorting (FAPS) and identified the components by liquid chromatography-tandem mass spectrometry (LC-MS/MS) analysis. We found that each phyB photobody is composed of thousands of phyB dimers and other proteins that can be classified into two groups based on their requirement for other factors to localize to the phyB photobody in protoplasts.

## Results

### Isolation of phyB photobodies

**Fig. 1:**
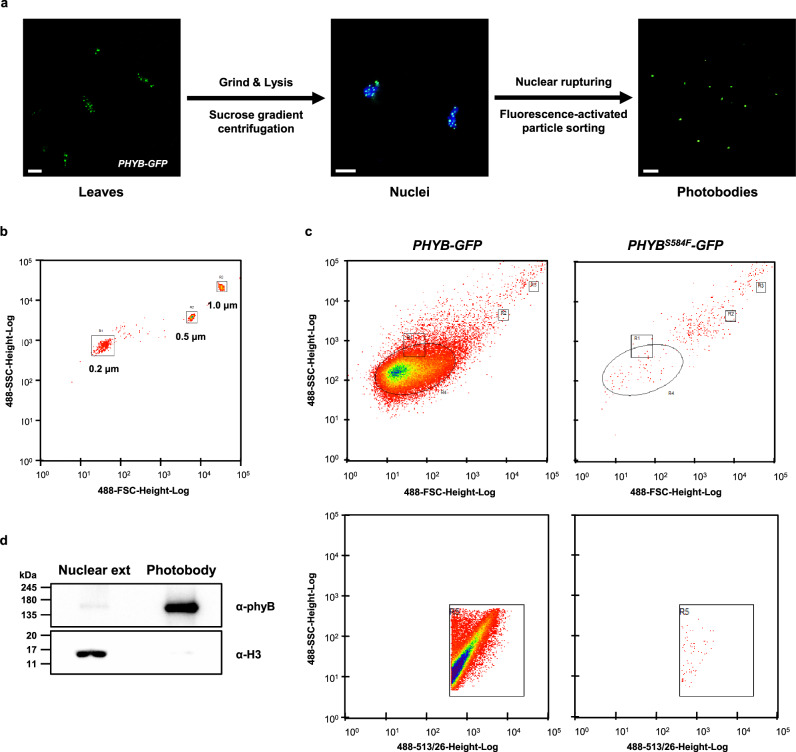
Isolation of phyB photobodies by fluorescence-activated particle sorting. **a** The isolation scheme for phyB photobodies. Mature leaves of *PHYB-GFP* are sampled (left) and ground and nuclei are isolated by sucrose gradient centrifugation (middle). The nuclei are ruptured by sonication and photobodies are isolated by fluorescence-activated particle sorting (right). Images are captured under confocal microscopy with (Nuclei) or without (Leaves, Photobodies) DAPI staining. Scale bars = 10 μm. **b** The forward and side-scatter parameters of 0.2-, 0.5-, and 1-μm reference beads (See Supplementary Fig. 1). The reference beads were sorted to estimate the parameters for the phyB photobody. Each red dot indicates the position of a sorted reference bead and the main position of each reference bead is indicated by a square. **c** The determination of a scatter gate and FITC gate. *PHYB-GFP* and *PHYB*^*S584F*^*-GFP* samples were sorted and the scatter gate (ellipse, upper panels) and the FITC gate (square, lower panels) were set to sort for particles present in large excess in the *PHYB-GFP* sample compared to the *PHYB*^*S584F*^*-GFP* sample. **d** Immunoblot analysis showing the enrichment of phyB but not histone H3 in the isolated photobody sample. Equal amounts (1.5 μg) of pre-sorted nuclear extract proteins (Nuclear ext) and sorted photobody proteins (Photobody) were immunoblotted for phyB and histone H3 using the corresponding antibodies (α-phyB, α-H3). The protein molecular weights of the SDS-PAGE-separated proteins are marked on the left and indicated with kDa. The protein amounts were quantified by the BCA protein assay. Source data are provided as a Source Data file.

We isolated phyB photobodies from mature *Arabidopsis* leaves using FAPS. The summarized workflow is shown in Fig. 1a. Transgenic plant leaves expressing phyB-GFP were harvested, frozen immediately with liquid nitrogen, and ground, and nuclei were isolated by sucrose gradient centrifugation. The isolated nuclei were ruptured to make the nuclear extract, and photobodies were isolated from the nuclear extract by FAPS.

We triggered the sorting by setting a relatively high threshold for green fluorescence signal and applying a scatter gate and a FITC gate. To determine the scatter gate, we roughly estimated the optical size of the photobody by comparing photobodies with green fluorescent flow cytometry size reference beads under fluorescence microscopy. We found that the optical sizes of photobodies were about 0.2 μm (Supplementary Fig. 1). We sorted reference beads to estimate their forward and side-scatter parameters (Fig. 1b). We then sorted the nuclear extracts of *PHYB-GFP* and the *PHYB*^*S584F*^*-GFP* plants to determine the gates. *PhyB*^*S584F*^-GFP displays only diffuse green fluorescence nuclear signal both in transgenic plants and in protoplasts (Supplementary Fig. 2). We set gates that covers green fluorescence particles present in large excess in the *PHYB-GFP* sample compared to the *PHYB*^*S584F*^*-GFP* sample (Fig. 1c). Using these gates, we were able to reproducibly sort particles enriched in the *PHYB-GFP* sample.

The isolated particles showed green fluorescence signals (Fig. 1a). To further confirm that the isolated particles were enriched for phyB photobodies, we performed an immunoblot assay with an anti-phyB antibody using equal amounts of input nuclear extract proteins and isolated particle proteins. The results showed that phyB was highly enriched in the isolated particles, whereas a nuclear protein, histone H3, was disenriched in the isolated particles (Fig. 1d). These results suggest that the established method selectively isolates particles that are highly enriched for phyB photobodies.

### Quantifying the phyB molecules in a photobody

Each photobody is likely to comprise many phyB molecules, considering its relatively large optical size. Thus, we used an immunoblot assay to determine the number of phyB molecules in an isolated photobody. For the assay, we estimated the amount of phyB protein in 10 million sorted particles by comparison with recombinant phyB protein as a reference (Supplementary Fig. 3). By assuming that all sorted particles are phyB photobodies, we estimated that a photobody contains about 3016 phyB protein molecules, which correspond to about 1500 phyB dimers. Since the size of phyB photobodies varies depending on the light conditions, temperature, and time of day^5,11–14,29^, the number of phyB molecules in a photobody is likely to be dynamic. Our estimated number of phyB molecules in a photobody is different from a previously estimated number based on the fluorescence intensity^11^, which may be caused by different experimental conditions and estimation methods. Thus, the calculated number of phyB molecules in a photobody should be taken as a rough estimate specific to our experimental conditions. With this caveat, our analyses suggest that a photobody contains about 1500 phyB dimers.

### The identification of phyB photobody components by LC-MS/MS

**Fig. 2:**
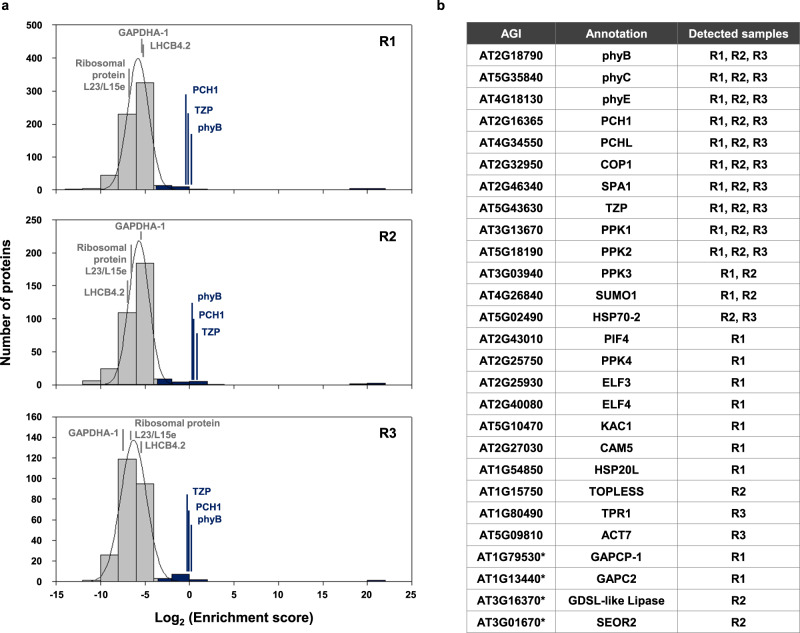
LC-MS-MS analysis of photobody components. **a** Histograms of normalized enrichment scores with fitted normal distribution curves for three independent samples (R1, R2, R3). The LFQ intensity of a protein was normalized by the LFQ intensity of phyB in each sample (normalized LFQ). The enrichment score of a protein is calculated by dividing the normalized LFQ of a protein in a sorted sample by the normalized LFQ of the same protein in the pre-sorted nuclear extract input sample. The distributions of logarithmic enrichment scores (Log_2_ (Enrichment score)) were approximately normal for all samples (R^2^ = 0.9993 for sample 1, 0.9971 for sample 2, 0.9980 for sample 3). Photobody components were defined as proteins whose Log_2_ (Enrichment score) was higher than μ + 2 σ value (dark blue bars) where μ is the average and σ is the standard deviation of the fitted normal distribution curve. The positions of phyB, PCH1, and TZP are indicated. **b** The candidate list of photobody components. Proteins that meet the criteria described in (**a**) were selected as the candidates for the phyB photobody components. Four proteins excluded from the candidates are indicated by asterisk (*). Source data are provided as a Source Data file.

To identify phyB photobody components, we analyzed both sorted and pre-sorted input samples by LC-MS/MS. The histogram of normalized enrichment scores was plotted and the average and standard deviation (σ) were calculated from the fitted normal distribution curve (Fig. 2a). Proteins having a normailzed enrichment score higher than 2σ from the average were selected as candidate phyB photobody components. We repeated the experiment three times with independently grown plant samples and selected candidates that satisfied the criterion at least once (Supplementary Fig. 4). Twenty-seven proteins, including phyB itself, were selected (Fig. 2b and Supplementary Data 1). From among them, we excluded two chloroplastic GAPDHs (GAPCP1 and GAPCP2), an apoplastic GDSL-like lipase, and a component of the sieve element protein body (SEOR2) based on their obviously different subcellular localizations. Thus, we identified 23 final candidates.

### The validations of phyB photobody components

Among the 23 final candidates, five proteins (PCH1, PCHL, COP1, SPA1, and TZP) had previously been identified as phyB photobody components^15,16,23,35–37,44^. The other candidate proteins had not previously been experimentally assessed for photobody localization. Thus, we systematically determined if the identified candidate proteins could localize to the phyB photobody by transiently expressing them in protoplasts made from transgenic plants expressing phyB-GFP. Hereinafter, unless otherwise specified, the term “protoplasts” refers to protoplasts made from transgenic plants expressing phyB-GFP. We experimentally determined the localization of all but one candidate protein in the list (Fig. 2b). The exception was SUMO1, which is a small ubiquitin-like modifier known to post-translationally modify phyB and PIF3^45,46^. We further included five additional family members of candidate proteins in our analysis. From our analysis, we found that most of the candidate proteins could localize to the phyB photobody, and could be classified into two groups based on their requirement for other proteins to localize to the phyB photobody in our experimental conditions.

**Fig. 3:**
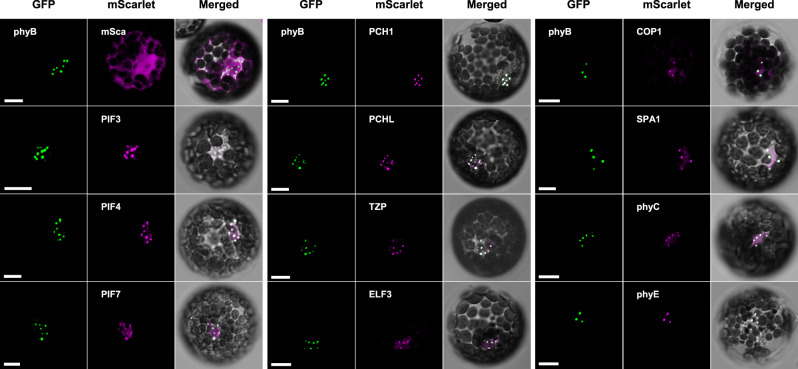
PhyB photobody components that readily localize to the phyB photobody. mScarlet-fused candidate proteins were transiently expressed in protoplasts prepared from *PHYB-GFP* transgenic plants. Images were taken under confocal microscopy using the GFP channel (488nm-525/25 nm) for phyB-GFP (GFP, green color) and the RFP channel (561nm-595/25 nm) for mScarlet-fused proteins (mScarlet, magenta color). The fluorescence images were merged with white light images (Merged). Non-fused mScarlet (mSca) was used as a control protein and showed diffuse mSca signals. The transformed protoplasts were cultured for 16 h under continuous white light (10 μmol/m^2^/s) before observation. Scale bars = 10 μm. The representative images were shown in observation of at least 30 independent protoplasts. The observed fractions of the protoplasts showing co-localization are listed in Supplementary Data 2. The quantitative fluorescent intensity profiles of the co-localization are in Supplementary Fig. 5. Source data are provided as a Source Data file.

The first group includes candidate proteins that localized to the phyB photobody more than 70% when their mScarlet (mSca)-tagged forms were transiently expressed in protoplasts without co-expression of other proteins (Fig. 3 and Supplementary Fig. 5). mSca alone showed diffuse red fluorescence signals, while phyB-GFP formed green fluorescence nuclear bodies in protoplasts. Coilin is known to form the nuclear Cajal body independent of the phyB photobody^47^. As expected, coilin-mSca formed red spots that did not overlap with the green phyB photobodies. In contrast, mSca-tagged PIF4 formed red spots that overlapped with the phyB photobodies. Among the PIF proteins, PIF4 alone was included in the final candidate list from the LC-MS/MS analysis. We hypothesized that this was due to the low abundances of other endogenous PIFs (Supplementary Fig. 6), and thus also determined the localizations of PIF3 and PIF7. As seen for PIF4, mSca-tagged PIF3 and PIF7 also formed red spots that overlapped with the phyB photobodies (Fig. 3). mSca-tagged PCH1, PCHL, TZP, ELF3, phyC, phyE, COP1, and SPA1 also formed red spots that overlapped with the phyB photobodies (Fig. 3). These results indicate that PIFs, PCH1, PCHL, TZP, ELF3, COP1, SPA1, phyC, and phyE are phyB photobody components that readily localize to the phyB photobody when transiently expressed in protoplasts. Notably, all proteins in the first group are phyB-interacting proteins^15,16,23,35,48–50^.

The second group includes candidate proteins that localize to the photobody less than 10% when expressed alone but localize to the photobody more than 70% when co-expressed with a member of the first group in our experimental conditions. Proteins in the second group could be further divided into two subsets depending on their required first-group members.

**Fig. 4:**
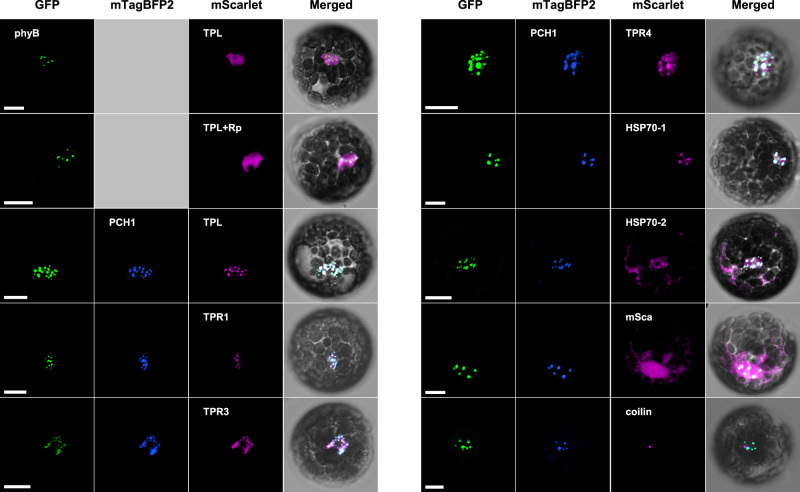
PhyB photobody components that localize to the phyB photobody when co-expressed with PCH1. mScarlet-fused candidate proteins were transiently co-expressed with mTagBFP2-fused PCH1 in protoplasts prepared from *PHYB-GFP* transgenic plants. Images were taken under a confocal microscope using the GFP channel (488 nm-525/25 nm) for phyB-GFP (GFP, green color), the BFP channel (405 nm-450/25 nm) for PCH1-mTagBFP2 (mTagBFP2, blue color), and the RFP channel (561 nm-595/25 nm) for mScarlet-fused proteins (mScarlet, magenta color). The fluorescence images were merged with white light images (Merged). Coilin-mScarlet was used as a control protein that is known to form a phyB-independent nuclear body. Rp indicates red light pulse (25 μmol/m^2^/s for 30 min) treated protoplast sample image to observe localization of the TPL under the high red to far-red light ratio. The transformed protoplasts were cultured for 16 h under continuous white light (10 μmol/m^2^/s). Scale bars = 10 μm. The representative images were shown in observation of at least 30 independent protoplasts. The observed fractions of the protoplasts showing co-localization are listed in Supplementary Data 2. The quantitative fluorescent intensity profiles of the co-localization are in Supplementary Fig. 7. Source data are provided as a Source Data file.

The members of the first subset localize to the phyB photobody when co-expressed with PCH1 (Fig. 4 and Supplementary Fig. 7). TOPLESS (TPL) is an example of this group. TPL and four TPL-RELATED (TPR) proteins (TPR1-TPR4) are WD40 repeat proteins belonging to the Groucho-type transcriptional corepressor family; they repress mRNA expression upon binding to various DNA-binding transcription factors^51^. Among the TPL family members, our LC-MS/MS analysis identified TPL and TPR1 as phyB photobody components (Fig. 2). The isolated photobodies were also enriched with TPL when detected with an anti-TPL antibody (Supplementary Fig. 8). We determined the photobody localization of TPL in protoplasts. When TPL-mSca alone was expressed in protoplasts, it formed diffuse red fluorescence signals rather than discrete red spots, while phyB-GFP clearly formed the green photobodies (Fig. 4). The irradiation of a red light pulse did not cause TPL to localize to the phyB photobodies (Fig. 4), suggesting that the diffuse red fluorescence signals of TPL-mSca was not due to a low ratio of Pfr/Pr. Since phyB photobodies could include proteins that are recruited not only by phyB but also by phyB-interacting proteins, we hypothesized that the co-expression of a first group member may facilitate the incorporation of TPL into the photobody by increasing the protein level of the first group member. We co-expressed mTagBFP2 (mBFP)-tagged PCH1, as the *pch1* mutation is associated with a major decrease in the size of the phyB photobody^15,16^. When TPL-mSca was co-expressed with PCH1-mBFP, TPL-mSca formed red fluorescent spots that co-overlapped with the green fluorescent spots of phyB-GFP and the blue fluorescent spots of PCH1-mBFP (Fig. 4). Similarly, mSca-tagged TPR1, TPR3 and TPR4 localized to the phyB photobody when co-expressed with PCH1-mBFP (Fig. 4). PhyB-GFP and PCH1-mBFP formed photobodies equally well both in wild type and *tpl/tpr1/tpr4* triple mutant (*tplT*) protoplasts (Supplementary Fig. 9), supporting that TPL/TPR family members are clients of phyB photobody components that are recruited to the phyB photobody by PCH1. HSP70-1 and its family member, HSP70-2, also localized to the phyB photobody when co-expressed with PCH1 (Fig. 4). Together, these results indicate that this subset of candidate proteins are phyB photobody components that localize to the phyB photobody when co-expressed with PCH1.

**Fig. 5:**
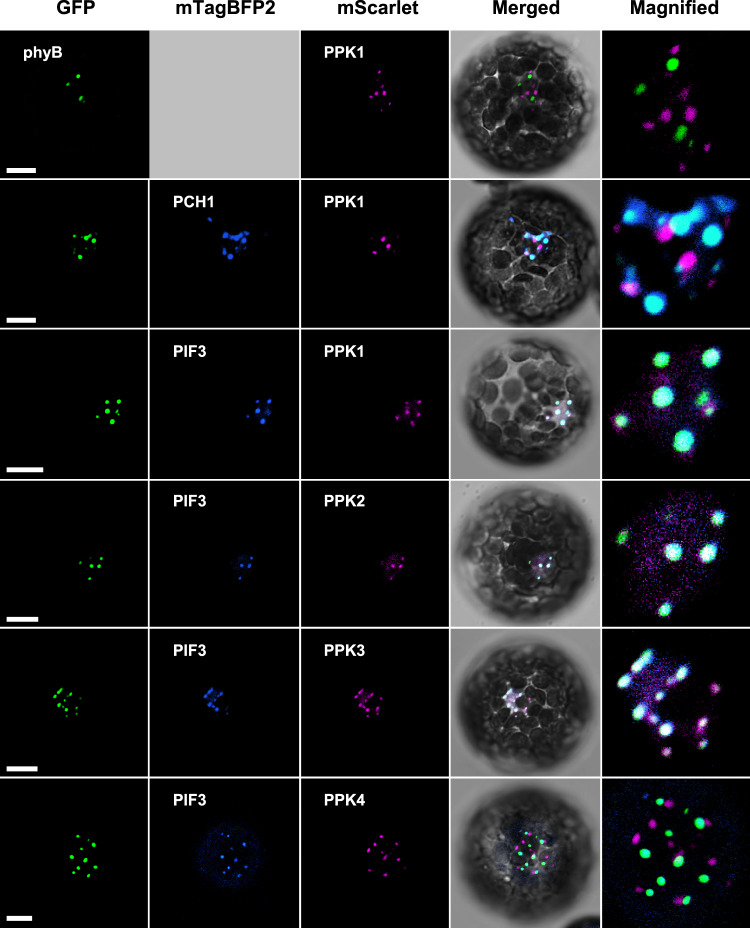
PhyB photobody components that localize to the phyB photobody when co-expressed with PIF3. mScarlet-fused PPKs were transiently co-expressed with mTagBFP2-fused PIF3 in protoplasts prepared from *PHYB-GFP* transgenic line. Images were taken under a confocal microscope using the GFP channel (488 nm-525/25 nm) for phyB-GFP (GFP, green color), the BFP channel (405 nm-450/25 nm) for PCH1- mTagBFP2 (mTagBFP2, blue color), and the RFP channel (561 nm-595/25 nm) for mScarlet-fused proteins (mScarlet, magenta color). The fluorescence images were either merged with white light images (Merged) or magnified (Magnified). An empty square indicates no co-transformation of any mTagBFP2-fused protein. The transformed protoplasts were cultured for 16 h under continuous white light (10 μmol/m^2^/s). Scale bars = 10 μm. The representative images were shown in observation of at least 30 independent protoplasts. The observed fractions of the protoplasts showing co-localization are listed in Supplementary Data 2. The quantitative fluorescent intensity profiles of the co-localization are in Supplementary Fig. 10. Source data are provided as a Source Data file.

The members of the second subset localize to the phyB photobody when co-expressed with PIF3. PPK family members belong to this subset. When expressed alone in protoplasts, PPK1-mSca formed red nuclear spots that do not overlap with the phyB-photobody (Fig. 5 and Supplementary Fig. 10). Unlike the proteins in the first subset, most of the red spots formed by PPK1-mSca did not overlap with the phyB photobodies even when co-expressed with PCH1-mBFP (Fig. 5), indicating that PPK1 mainly forms phyB-independent nuclear bodies when expressed alone or co-expressed with PCH1 in protoplasts. Since PPK1 binds to PIF3^24^, we examined if the co-expression of PIF3-mBFP could recruit PPK1-mSca to the phyB photobody. Indeed, PPK1-mSca formed red spots that overlapped with the phyB photobody when co-expressed with PIF3-mBFP (Fig. 5). PPK2-mSca and PPK3-mSca also localized to the phyB photobody when co-expressed with PIF3, whereas most of the red nuclear spots corresponding to PPK4-mSca still did not overlap with the phyB photobody even when co-expressed with PIF3 (Fig. 5). Together, these results indicate that PPK1, PPK2, and PPK3 are phyB photobody components that localize to the phyB photobody when co-expressed with PIF3. Unlike the other components, however, PPKs can also localize to nuclear bodies other than the phyB photobody (see Discussion).

The remaining candidates included proteins that did not localize to the phyB photobody in our experimental conditions. ACT7, CAM5, ELF4, HSP20L, and KAC1 with N-terminal or C-terminal mSca tags showed diffuse red signals when expressed either alone or together with PCH1-mBFP (Supplementary Fig. 11a). ELF4 is known to form an evening complex with ELF3 and LUX^52^. However, we could not observe the localization of ELF4 to the photobody even when co-expressed with ELF3 in our experimental condition (Supplementary Fig. 11b). These results suggest that ACT7, CAM5, ELF4, HSP20L, and KAC1 are either false positives erroneously identified by the method or photobody components that require the co-expression of yet other proteins for their localization to the phyB photobody.

### TPL/TPR interact with PCH1 and regulate hypocotyl elongation

Among the examined components, HSP70s and TPL/TPRs are photobody components that have not been previously implicated in phyB signaling other than TPL being listed as one of proteins co-immunoprecipitated with PCH1 (Supplementary Fig. 12)^15^. Of them, we further analyzed TPL/TPR family members to examine whether the phyB photobody components identified by our method regulate phyB signaling.

**Fig. 6:**
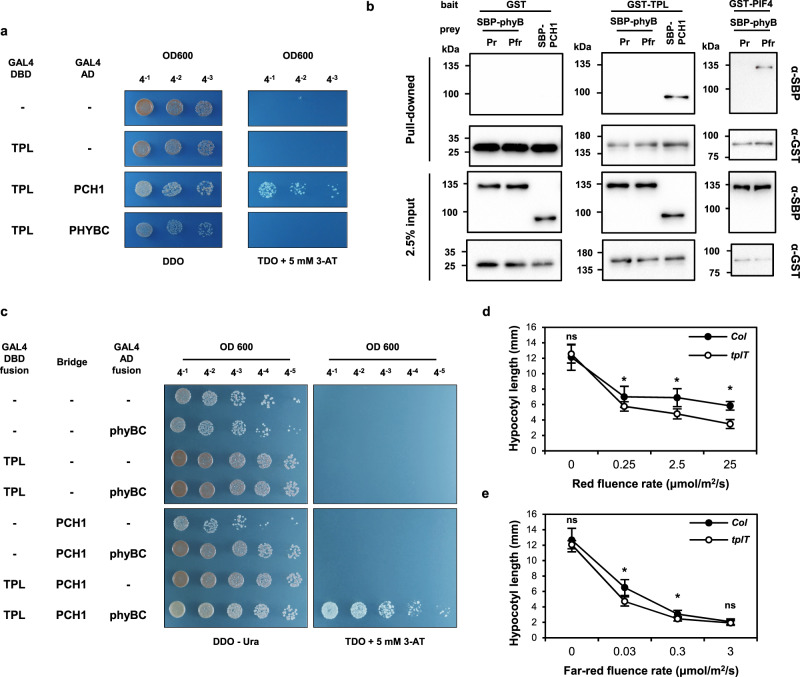
TPL/TPR family members are PCH1-interacting phyB photobody components that regulate hypocotyl elongation. **a** Yeast two hybrid assay showing the interaction between TPL and PCH1 but not between TPL and the C-terminal domain of phyB (phyBC) in a yeast two-hybrid assay. The assay was performed with TPL fused to the GAL4 DNA binding domain (GAL4 DBD) and PCH1 and phyBC fused to the GAL4 transactivation domain (GAL4 AD). DDO: double drop-out synthetic medium lacking leucine and tryptophan. TDO: triple drop-out synthetic media lacking histidine, leucine, and tryptophan. 3-AT: 3-amino-1,2,4-triazole. Yeast transformants selected on DDO were serially diluted (OD600) and spotted on DDO and TDO with 5 mM 3-AT. Empty vectors are marked as “-”. **b** In vitro pull-down assay showing the interaction between TPL and PCH1 but not between TPL and phyB. GST and GST-fused TOPLESS (GST-TPL) or PIF4 (GST-PIF4) were incubated with SBP-fused PCH1 (SBP-PCH1) or phyB (SBP-phyB) and pulled down with glutathione resin. Both input (2.5% input) and pulled down (pull-down) proteins were detected with either anti-GST antibody (α-GST) or anti-SBP antibody (α-SBP). The Pr or Pfr form of phyB was obtained by applying a far-red light pulse (3 μmol/m2/s, 10 min) or a red light pulse (25 μmol/m^2^/s, 10 min) before the incubation; all subsequent procedures were done under a green safety light. **c** Yeast three hybrid assay showing the interaction between phyBC and TPL in the presence of PCH1. The assay was performed similarly to the yeast two hybrid assay except PCH1 was additionally expressed under the control of constitutive ADH1 promoter in a bridge vector. Empty vectors are marked as “-”. Uracil was dropped out for an auxotrophic marker for the bridge vector. **d**, **e** Fluence response curves for hypocotyl elongation of *tpl/tpr1/tpr4* (*tplT*) triple mutant seedlings. Wild type (Col) and the *tplT* mutant were grown under varying fluence rates of continuous red light (**d**, 0 to 25 μmol/m^2^/s) or far-red light (**e**, 0 to 3 μmol/m^2^/s) for 4 days. Data are presented as mean values +/− SD (*n* ≥ 29 seedlings). An asterisk (*) indicates statistically significant difference (*p* < 0.0001, two-tailed; ns not significant). Source data are provided as a Source Data file.

We speculated that PCH1 may directly interact with TPL and recruit it to the phyB photobody. Indeed, our yeast two-hybrid assay showed that TPL interacts with PCH1 (Fig. 6a). GST-fused recombinant TPL protein also interacted with recombinant PCH1 protein, whereas GST alone did not, supporting the notion that TPL is a PCH1-interacting protein (Fig. 6b). In contrast, a yeast two-hybrid assay showed that TPL does not interact with the C-terminal domain of phyB (Fig. 6a). Recombinant TPL also failed to interact with recombinant full-length phyB in the Pfr or Pr form in an in vitro binding assay, whereas the positive interaction control, PIF4, interacted preferentially with the Pfr form of phyB (Fig. 6b). A yeast three hybrid assay further showed that phyB interacts with TPL only when PCH1 is co-expressed (Fig. 6c), supporting that PCH1 bridges the interaction between phyB and TPL. HSP70-1 belonging to the same subset of the second group with TPL also interacted with PCH1 but not with phyB (Supplementary Fig. 13). Together, these results imply that TPL is recruited to the phyB photobody not directly by phyB but by phyB-interacting PCH1.

We next investigated whether TPL/TPR family members could regulate hypocotyl elongation in red or far-red light. For this analysis, we grew *tpl/tpr1/tpr4* triple mutant (*tplT*) seedlings under different fluence rates of red and far-red lights and determined hypocotyl lengths. We found that *tplT* mutant seedlings have shorter hypocotyl lengths than wild type seedlings in all red fluence rates we examined (Fig. 6d), while the mutant has mildly shorter hypocotyl lengths only in low fluence rates of far-red light (Fig. 6e). The shorter hypocotyl lengths were not due to a general growth defect, as the mutants had hypocotyl lengths similar to those of the wild type when grown in the dark. Taken together, these results indicate that the method identified photobody components that regulate hypocotyl elongation both in red and far-red light.

## Discussion

Here, we report a FAPS-based method for isolating phyB photobodies. The isolation method consists of isolating nuclei from ground leaf samples via sucrose gradient centrifugation, rupturing the nuclei by sonication, and then sorting phyB photobodies with FAPS. The isolated phyB photobodies were highly enriched for phyB, containing about 1500 phyB dimers per photobody, but were devoid of a nuclear protein, histone H3. Our LC-MS/MS analysis of the isolated photobodies identified 23 highly enriched proteins, including phyB itself. The list significantly overlaps with proteins co-immunoprecipitated with ELF3 or PCH1 (Supplementary Fig. 12)^15,53^. The reported co-immunoprecipitated proteins include photobody components that do not directly interact with ELF3 or PCH1, suggesting that phyB photobodies might also have been precipitated by ELF3 and PCH1. The majority of the identified proteins and their homologs localized to the phyB photobody when expressed in protoplasts, demonstrating the utility of our isolation method for identifying phyB photobody components. The TPL/TPR family members were among identified components by our method. Our analysis of the *tpl/tpr1/tpr4* triple (*tplT*) mutant showed that TPL/TPR family members negatively regulate the red light-induced inhibition of hypocotyl elongation, further demonstrating that our method identified photobody components that regulate hypocotyl elongation in red light. The *tplT* mutant also has mildly shorter hypocotyl length than wild type in low fluence rates of far-red light. Since PCH1 does not interact with phyA^15^, the results suggest that TPL/TPR family members regulate phyA signaling independent of PCH1.

The phyB photobody is a membraneless organelle formed by LLPS^11^. Such membraneless organelles are typically made of a scaffold protein and its interacting client proteins^54^. Our analysis indicates that the phyB photobody is also made of a scaffold protein and client proteins, with phyB as the main scaffold protein, phyB-interacting proteins as the primary clients, and primary client-interacting proteins as the secondary clients. In other words, the phyB photobody is made of phyB and other proteins that could be classified into two groups based on their requirement for other components to localize to the phyB photobody in our experimental conditions. The first group includes COP1/SPA1, phyC/phyE, PCH1/PCHL, PIF3/PIF4/PIF7, ELF3, and TZP, which readily localize to the phyB photobody when expressed alone in protoplasts (Fig. 3). All these components directly interact with phyB^15,16,23,35,48–50,55^, suggesting that they are primary clients that are recruited to the photobody directly by phyB itself. The interaction between primary clients such as COP1 and SPA1, COP1 and ELF3, and PCH1 and PIF1 may also contribute their recruitments to the phyB photobody^8,56,57^. The second group includes proteins that interact with a member of the first group rather than phyB and localize to the phyB photobody when co-expressed with a member of the first group (here, PCH1 and PIF3). Among them, HSP70-1/HSP70-2 and TPL/TPR1/TPR3/TPR4 localize to the phyB photobody when co-expressed with PCH1 (Fig. 4). Our analysis shows that HSP70-1 and TPL interact with PCH1 but not phyB (Fig. 6 and Supplementary Fig. 13), suggesting that HSP70-1 and TPL are recruited to the photobody not directly by phyB but via its primary client, PCH1. The PIF3-requiring components include PPK family members (PPK1 to PPK3; Fig. 5). PPK1 interacts strongly with PIF3 but only very weakly with phyB in a light-independent manner^24^, suggesting that PPKs are recruited to the photobody largely through their strong interactions with the primary client, PIF3. Together, our results support the idea that the phyB photobody comprises phyB, primary clients that interact with phyB, and secondary clients that interact with the primary clients.

Curiously, we noted that PPKs form nuclear bodies that do not overlap with the phyB photobody when expressed alone (Fig. 5), indicating that PPKs can participate in different nuclear bodies depending on the presence of their interacting proteins. Consistent with their localization in different nuclear bodies, PPKs, which are also known as MUT9P-LIKE KINASES 1-4 (MLK1-MLK4), regulate not only phyB signaling but also other processes, such as ABA signaling, blue light signaling, and flowering. PPKs regulate these processes by phosphorylating various target proteins. Their targets include histone H3, the phosphorylation of which leads to the transcriptional repression of the loci^31,58^, and other proteins, such as PIF3^24^, the ABA receptors PYR/PYLs^59^, the blue light photoreceptor CRY2^60^, and the PAR-binding transcriptional corepressor RCD1^32^, the phosphorylations of which lead to the degradation of these target proteins. PPKs have been also shown to interact with ELF3, which forms the evening complex with ELF4 and LUX^53^. Since CRY2 is reported to form nuclear bodies^61^, we examined if PPK1 nuclear bodies overlapped with CRY2 nuclear bodies in protoplasts. Interestingly, when PPK1 and CRY2 were co-expressed in protoplasts, the majority of PPK1 nuclear bodies overlapped with CRY2 nuclear bodies, but did not greatly overlap with phyB photobodies (Supplementary Fig. 14). These results suggest that PPKs shuttle between the phyB photobody and the CRY2 photobody depending on the presence of PIF3. Notably, a few PPK1 nuclear bodies overlap with neither phyB photobodies nor CRY2 photobodies (Supplementary Fig. 14). Further studies are needed to determine the identity of these photobody-independent PPK1 nuclear bodies.

Our identification of HSP70 family members as phyB photobody components supports the idea that chaperone proteins are a common ingredient of many membraneless organelles. Previous studies showed that various chaperones, such as HSP40, HSP70, and HSP90, are components of stress granules, which are cytosolic membraneless organelles that store RNA molecules stalled at the pre-initiation complex stage in yeast, fruit fly, and mammalian cells^62^. Human HSP70 family members, including HSPA1A and HSPA8, are recruited to nuclear bodies by the RNA binding protein, TDP-43 (TAR DNA-binding protein 43 kDa), in human neuronal cells^63,64^. Plant cytosolic condensates formed by NPR1 or Rbp47b also include HSP70 members^65,66^. This recruitment of HSPs to membraneless organelles has been proposed to influence the assembly and disassembly of membraneless organelles and prevent the bodies from undergoing pathological aggregation^64,67^. In light of these previous findings, we speculated that HSP70s could stabilize the phyB photobody by preventing non-specific aggregation.

The photobody isolation method we established herein is likely to include false positives. Thus, the candidate proteins must be validated for their photobody localization by other means. To reduce false positive proteins, we applied a relatively strict enrichment score criterion to select the candidate proteins from the LC-MS/MS data. Nevertheless, the list included two chloroplastic GAPDHs, an apoplastic lipase, and a sieve element protein body component. The list also included ACT7, CAM5, ELF4, HSP20L, and KAC1, which did not localize to the phyB photobody when co-expressed with PCH1 or ELF3 in protoplasts (Supplementary Fig. 11). Some of these proteins may have been selected as candidates because of our assignment of 1 input LFQ value for proteins having 0 input LFQ value to avoid the division by zero. However, we should not exclude the possibility that these proteins might localize to the photobody if co-expressed with another appropriate protein. The method we used is also likely to miss some true positives, such as due to the detection limits of the spectrometry. Thus, the list of phyB photobody components presented here should not be taken as representing all photobody components. For example, we found that PIF3 and PIF7 could localize to the phyB photobody when expressed in protoplasts (Fig. 3), even though our method identified only PIF4 as a photobody component among the PIF family members (Fig. 2b). Similarly, HSP70-1, TPR3 and TPR4, which are homologs of identified HSP70-2 and TPL, respectively, also localized to the phyB photobody when expressed in protoplasts (Fig. 4). Beyond these family members, other factors previously shown to localize to the photobody were not identified in our analysis, including BBX4, RRC1, SFPS, and SMP2^39,41–43^. SPA family members in addition to SPA1 are also expected to be present in the photobody, as they interact with phyB and COP1^68^. The use of more photobodies for LC-MS/MS analysis might facilitate the identification of rare photobody components. Since the composition of photobody components is expected to be dynamic depending on the time of day and environmental conditions, the sampling of plants at different times of day or in different environmental conditions may also facilitate the identification of dynamically changing photobody components.

The list of photobody components is consistent with previously suggested roles of photobody. First, the photobody includes the ‘dark factors’, COP1 and SPA1 that inhibit light responses in the dark, by degrading various proteins including HY5 and HFR1^8^. PhyB inhibits the COP1/SPA complex either by protein degradation (SPAs), nuclear exclusion (COP1), or the disorganization of the complex^8^. The capturing of COP1/SPA in the photobody would add another layer of inhibition by separating the complex from its target proteins. The photobody also includes other dark factors, PIFs. PhyB inhibits PIFs either by protein degradation, sequestration, or masking of activation domains^2,69^. Since the thousands of PIFs’ target promoters spread throughout the entire genome are not all likely to be in a few photobodies^70^, the capturing of PIFs in the photobody could also be another layer of inhibition by separating PIFs from their target promoters. Second, the photobody includes PPKs that have been shown to phosphorylate both light-activated phyB and PIF3^24^, which are subsequently ubiquitinated and degraded. Interestingly, our transient expression analysis indicates that PPKs are recruited to the photobody when co-expressed with PIF3 (Fig. 5). This suggests that PIF3 may be phosphorylated in the photobody, consistent with the notion that it is where PIFs are phosphorylated and subsequently ubiquitinated for degradation. Third, the phobody includes TZP whose photobody formation was shown to be disrupted by chemicals inhibiting active transcription^23^, indicating that the photobody may have a role in transcription. Interestingly, identified components include transcription corepressors, TPL/TPRs, although further studies are needed to confirm whether TPL/TPRs act as transcription corepressors or if they are sequestered in the photobody. Finally, the role of photobody could be further clarified as more components are identified. The number of the detected peptides in our current analyses are relatively modest (see Method section), suggesting that the list of photobody components likely remains incomplete. Photobodies isolated at different times of day or in different environmental conditions are likely to contain different components. The isolation of photobodies from different plant species may also add new components that suggest other roles of photobody. The methods established in this study will prove useful to identify other photobody components and to further elucidate the role of photobody.

## Methods

### Plant materials and growth conditions

*Arabidopsis thaliana* plants were grown at 22 °C in a growth room with a long-day light-dark cycle (16 h of white light (100 μmol/m^2^/s) and 8 h of dark). *PHYB* overexpression lines (*PHYB-GFP*) were generated by cloning a coding sequence of PHYB into a pCAMBIA-derived vector with the smGFP gene, transforming the construct into *phyB-9*, and selecting homozygous lines^71^. The primers used for cloning are listed in Supplementary Data 3.

### Fluorescence-activated particle sorting (FAPS)

FAPS were performed as previously described with modifications^72^. For photobody isolation, *PHYB-GFP/phyB-9* or *PHYB*^*S584F*^*-GFP/phyB-9*^71,73^ plants were grown at 22 °C in a growth room with a long-day light-dark cycle (16 h of white light (100 μmol/m^2^/s) and 8 h of dark) for 28 days. Mature rosette leaves (7 g) were sampled at Zeitgeber time (ZT) 8 and flash-frozen using liquid nitrogen.

The frozen sample was finely ground with a mortar and pestle, resuspended in 20 mL of buffer 1 (10 mM Tris, 10 mM MgCl_2_, 0.4 M sucrose, 160 μM MG132, 1 mM PMSF, and a protease inhibitor cocktail (Roche cOmplete^TM^), pH 8.00 titrated by HCl), and filtered through two layers of Miracloth (Merck, 475855). The pellet was collected by low-speed centrifugation (4 °C, 2500 g for 20 min) and washed 5 times with 15 mL of buffer 2 (10 mM Tris, 10 mM MgCl_2_, 1% (v/v) Triton X-100, 0.25 M sucrose, 160 μM MG132, 1 mM PMSF, and a protease inhibitor cocktail (Roche cOmplete^TM^), pH 8.00 titrated by HCl; 4 °C, 2500 g for 3 min). The pellet was resuspended in 400 μL of buffer 2 and layered onto 1 mL of buffer 3 (10 mM Tris, 2 mM MgCl_2_, 0.15% (v/v) Triton X-100, 1.7 M sucrose, 160 μM MG132, 1 mM PMSF, and a protease inhibitor cocktail (Roche cOmplete^TM^), pH 8.00 titrated by HCl) for sucrose gradient centrifugation (4 °C, 15,000 g for 30 min). The pellet was resuspended in 400 μL of buffer 2 and the sucrose-gradient centrifugation was repeated. The pelleted nuclei were incubated in 1 mL of buffer 4 (PBS with 0.2% (v/v) Triton X-100 and 1% (w/v) BSA, 160 μM MG132, 1 mM PMSF, and a protease inhibitor cocktail (Roche cOmplete^TM^), pH 8.00 titrated by HCl) for 1 h on a rotary shaker and collected by centrifugation (4 °C, 1500 g for 3 min). The nuclei were resuspended in 1 mL of buffer 4 with 10 μL of Alexa Fluor 488-conjugated anti-GFP antibody (Invitrogen, A-21311) and incubated for 16 h on a rotary shaker. The nuclei were collected by centrifugation (4 °C, 1500 g for 3 min) and washed three times with 1 mL of buffer 5 (PBS with 0.2% (v/v) Triton X-100, 160 μM MG132, 1 mM PMSF, and a protease inhibitor cocktail (Roche cOmplete^TM^), pH 8.00 titrated by HCl). The nuclei were washed and then ruptured by a sonicator (Bioruptor, B01020001) using the high-power output setting for 15 cycles of 30 sec On / 30 sec Off. The debris was removed by centrifugation (4 °C, 1500 g for 3 min). The supernatant was retrieved and adjusted to 2.1 mL with buffer 5, and 100 μL of the sample was set aside as the input sample for LC-MS/MS. The remaining 2 mL supernatant was sorted by fluorescence-activated particle sorting (FAPS).

Photobodies were isolated with a MoFlo Astrios Cell Sorter (Beckman Coulter) equipped with a nozzle size of 70 μm working at 60 psi pressure. Particles emitting strong green fluorescence were triggered to be sorted by setting 488-513/26 nm (excitation 488 nm, emission 513/26 nm) at a threshold of 0.3% and applying a 488-FSC-H-Logx488-SSC-H-Log gate (FSCxSSC) gate and a 488-513/26-H-Logx488-FSC-H-Log (FITCxFSC). To establish a FSCxSSC gate, the forward and side-scatter parameters were estimated based on comparison to particle size reference calibration beads (Invitrogen, F13839). The gate was set to include particles present in large excess in the *PHYB-GFP/phyB-9* sample compared to the *PHYB*^*S584F*^*-GFP/phyB-9* sample. The purity mode (1 envelope) and the differential pressure (0.4 to 0.5 psi) were used for the sorting. The photobody sorting efficiency was 86 to 95%. The sorting was repeated with three independently grown plant samples, resulting in 17.6, 13.2 and 15.1 million particles corresponding to 2.0, 1.4 and 1.8 μg of protein mass, respectively.

### LC-MS/MS

The LC-MS/MS analysis was performed using an RSLCnano u3000 / Orbitrap Exploris 240 (Thermo) system. The sorted photobodies and the input sample were collected and dried with a SpeedVac. The half of isolated particles (1.0, 0.7 and 0.9 μg of protein mass for three biological replicates) were used for the analysis. The dried samples were resuspended in 5.8 μL of lysis buffer (5% SDS, 50 mM TEAB, pH 8.5), reduced, alkylated, and digested with 1 μg trypsin/lysC (Pierce, A40009) on an S-trap micro (PROTIFI, C02-micro-80) for 1 h at 47 °C, according to the manufacturer’s protocol (v4.7). After digestion, peptides were eluted, dried using a Speedvac, and dissolved in 12 μL of 2% acetonitrile / 0.1% formic acid (Millipore). The dissolved peptides (5 μL) were loaded onto a trap column (Acclaim PepMap 100, 164535) and separated by analytical column (PepMap RSLC, ES802). The mobile phases were water with 0.1% formic acid (buffer A) and acetonitrile with 0.1% formic acid (buffer B). The following gradient of buffer B was applied at a flow rate of 300 nL/min (% buffer B): 2% to 20% over 100 min, and 20% to 32% over 20 min. The column temperature was set to 50 °C.

The survey scan settings were as follows: Resolution = 120,000, Max IT = auto, AGC 300%, mass range 350–1200 Th. The selected precursor was fragmented by HCD and analyzed by an Orbitrap. Other parameters for the MS/MS scan were as follows: Top15 double play, Resolution = 30,000, max IT = 200 ms, Threshold 1E5, normalized collision energy = 30%, isolation width = 2.0, dynamic exclusion parameter after *n* times = 1, exclusion duration time = 45 sec, mass tolerance low/high = 10 ppm. Raw data from the LC-MSMS were analyzed with Maxquant v1.6.10.43 and Perseus v1.5.8. The utilized Maxquant parameters were as follows:　database = UniProt Arabidopsis thaliana, enzyme = trypsin/P, variable modification = Oxidation (M), Acetyl (protein N-term), fixed modification = methylthio (C), LFQ (Label-Free quantification), and match between runs. The obtained ProteinGroup.txt file was filtered for ‘razor+unique peptide > = 2’ using Perseus. The number of the detected peptides in three biological replicates were 13,149, 8625 and 6823 for inputs and 4493, 1789 and 1201 for sorted particles.

### Recombinant protein purification

*PIF4*, *HSP70-1*, *PCH1* and *TOPLESS* were cloned into a pET41-derived expression vector to produce the N-terminal glutathione S-transferase (GST)-tagged and C-terminal His-tagged proteins. *PHYB* was cloned into a pBAD-derived expression vector to produce the N-terminal streptavidin binding peptide (SBP)-tagged and C-terminal His-tagged protein. PCH1 was cloned into a pET41-derived expression vector to produce the N-terminal SBP-tagged and C-terminal mTagBFP2-His-tagged protein. The pET41-derived vectors were transformed into *E. coli* BL21(DE3)-CodonPlus-RIL and pBAD-derived vectors were transformed into phycocyanobilin-producing LMG194^74^. Recombinant proteins were purified using Ni-NTA agarose (Qiagen Mat, 1018244) according to the manufacturer’s guidelines. The proteins were eluted with 250 mM imidazole and purified with glutathione-conjugated agarose resin or Streptavidin sepharose resin, according to the manufacturer’s guidelines (Cytiva 17-0756-01, Cytiva 17-5113-01). The eluted proteins were subjected to buffer exchange with a standard minimal buffer (50 mM Tris, 150 mM NaCl, 10% (v/v) glycerol, 0.05% (v/v) polysorbate 20, titrated by HCl to pH 7.40) using Amicon gravity concentration columns (EMD Millipore, UFC801024). The primers used for cloning are listed in Supplementary Data 3.

### In vitro pull-down assay

To determine the interaction between HSP70-1 or TPL and PCH1 or phyB, we performed an in vitro pull-down assay. GST-fused HSP70-1, TPL or GST-fused PIF4 were incubated with SBP-fused PCH1 or SBP-fused phyB in pull-down binding buffer (25 mM HEPES, 150 mM KCl, 1 mM DTT, titrated with KOH to pH 7.40) for 30 min at room temperature in the dark on a rotary shaker. Red (25 μmol/m^2^/s) or far-red light (3 μmol/m^2^/s) was irradiated for 10 min to convert phyB to Pfr or Pr, respectively, before the incubation. The complexes were pulled down using glutathione affinity gel (Cytiva, 17-0756-01). Samples were handled under safety green light. The pull-down samples were resolved by SDS-PAGE and transferred to a nitrocellulose membrane (GE Healthcare, 10600003). Immunoblotting was performed and antibody-bound target proteins were visualized with an ECL substrate (BioRad Cat, 1705062) and a ChemiDoc XRS + Imager.

### Yeast two-hybrid assay

To determine the interaction between TPL and PCH1 or the C-terminal domain of phyB (642 −1172 a.a.) in yeast, we performed a yeast two-hybrid assay according to the standard protocol (Clontech, PT3024-1). *TPL* was cloned into a pGBKT7 vector (pGBKT7-TPL) and *PCH1* and the C-terminal domain of *PHYB* were cloned into pGADT7 (pGADT7-PCH1, -PHYBC). *PCH1* was also cloned into p416ADH (p416ADH-PCH1). The two vectors were co-transformed into the AH109 yeast strain and plated on CSM dropout media lacking tryptophan and leucine (Double dropout media; DDO). The transformed yeast colonies were harvested and cultured in liquid DDO medium overnight. Yeast cells were collected by centrifugation (2000 g for 10 min) and washed three times with sterile water, and the optical density was adjusted to 0.25. Serial dilutions were prepared, and 10 μL of each serial dilution sample was spotted on DDO plates or dropout medium plates lacking tryptophan, leucine, and histidine (Triple dropout media; TDO) supplemented with 5 mM 3-aminotriazole AT. The plates were incubated at 30 °C for 3 days, and growth was visually observed. Yeast three-hybrid assay was similarly performed with pGBKT7-TPL, pGADT7-PHYBC, p416ADH-PCH1. Yeast three-hybrid assay was performed using URA3-deleted AH109.

### Transient expression in protoplasts

For transient expression, target genes were cloned into a pBI221-derived vector designed to express C-terminal mScarlet-fused or mTagBFP2-fused target proteins. The primers used for cloning are listed in Supplementary Data 3. Protoplasts were prepared as described previously^75^ from *PHYB-GFP* transgenic plants expressing phyB-GFP in the *phyB-9* mutant background under control of the 35 S promoter. Vectors were transformed as described previously and incubated at 22 °C for 18 h under continuous white light (10 μmol/m^2^/s).

### Confocal laser scanning microscopy

Confocal microscope images were obtained on the single focal plane (A1 HD25, Nikon) with the following laser settings: GFP (the excitation 488 nm and the emission 500 nm to 550 nm), mScarlet (the excitation 561 nm and the emission 570 nm to 620 nm) and mTagBFP2 (the excitation 405 nm and the emission 425 nm to 475 nm). The co-localizations were further visualized by fluorescence intensity profiles. All protoplast imaging experiments were obtained from at least 30 protoplasts and the quantified data were summarized in Supplementary Data 2.

### Hypocotyl length measurement

To measure hypocotyl length under different light conditions, sterilized seeds of Col and *tplT (tpl/tpr1/tpr4)* strains were plated on half strength MS agar plates and imbibed for 3 days in the dark at 4 °C. The plates were then incubated for 4 days in the dark or under varying fluence rates of red lights (0.25, 2.5, or 25 μmol/m^2^/s) or far-red light (0.03, 0.3, or 3 μmol/m^2^/s). Hypocotyl lengths were measured from more than 30 seedlings for each sample.

### Statistics and reproducibility

Differences in hypocotyl lengths were tested using two-tailed Student’s *t*-tests (Fig. 6d, e). The significance of the overlapping of two gene sets was tested using hypergeometric test (Supplementary Fig. 12). Figures show representative results from two independent experiments (Figs. 1d, 6b, Supplementary Fig. 13), 15 independent images (Supplementary Fig. 1 left; *PHYB-GFP*), 11 independent images (Supplementary Fig. 1 right; Reference beads), 40 independent images (Supplementary Fig. 2a, *PHYB-GFP* WLc), 5 independent images (Supplementary Fig. 2a, *PHYB-GFP* FRp), 18 independent images (Supplementary Fig. 2a, *PHYB*^*S584F*^*-GFP* WLc), 10 independent images (Supplementary Fig. 2a, *PHYB*^*S584F*^*-GFP* FRp), 3 biological replicates (Supplementary Fig. 2b), an experiment (Supplementary Figs. 2d, 6, 8), 3 independent experiments (Supplementary Fig. 3a). Protoplast transient expression images are representative results from at least 30 protoplasts.

### Reporting summary

Further information on research design is available in the Nature Portfolio Reporting Summary linked to this article.

## Supplementary information

Supplementary Information

Description of Additional Supplementary Files

Supplementary Data 1

Supplementary Data 2

Supplementary Data 3

Reporting Summary

## Data Availability

Source data are provided with this paper. Proteomics data have been deposited in PRIDE and the accession code is PXD040702. Source data are provided with this paper.

## Source data

Source Data
