# Supplementary material for: Phytochrome B photobodies are comprised of phytochrome B and its primary and secondary interacting proteins
Source: Nat Commun. 2023 Mar 27;14:1708. doi: 10.1038/s41467-023-37421-z (PMC10042835; doi:10.1038/s41467-023-37421-z)
Supplement: Supplementary file 1 — Supplementary Information [file 41467_2023_37421_MOESM1_ESM.pdf]

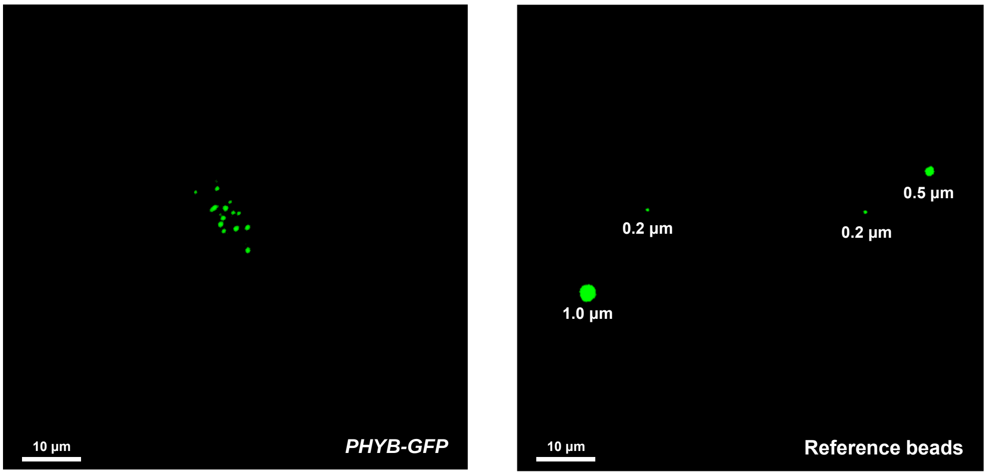

Supplementary Fig. 1 Optical sizes of phyB photobodies are similar to those of 0.2 μm size reference beads. Photobodies in a leaf epidermal cell of the *PHYB-GFP* transgenic line (left) and green fluorescent flow cytometry size reference beads (right) were observed under a confocal microscope using the GFP channel (488nm-525/25nm) with same gain parameters.

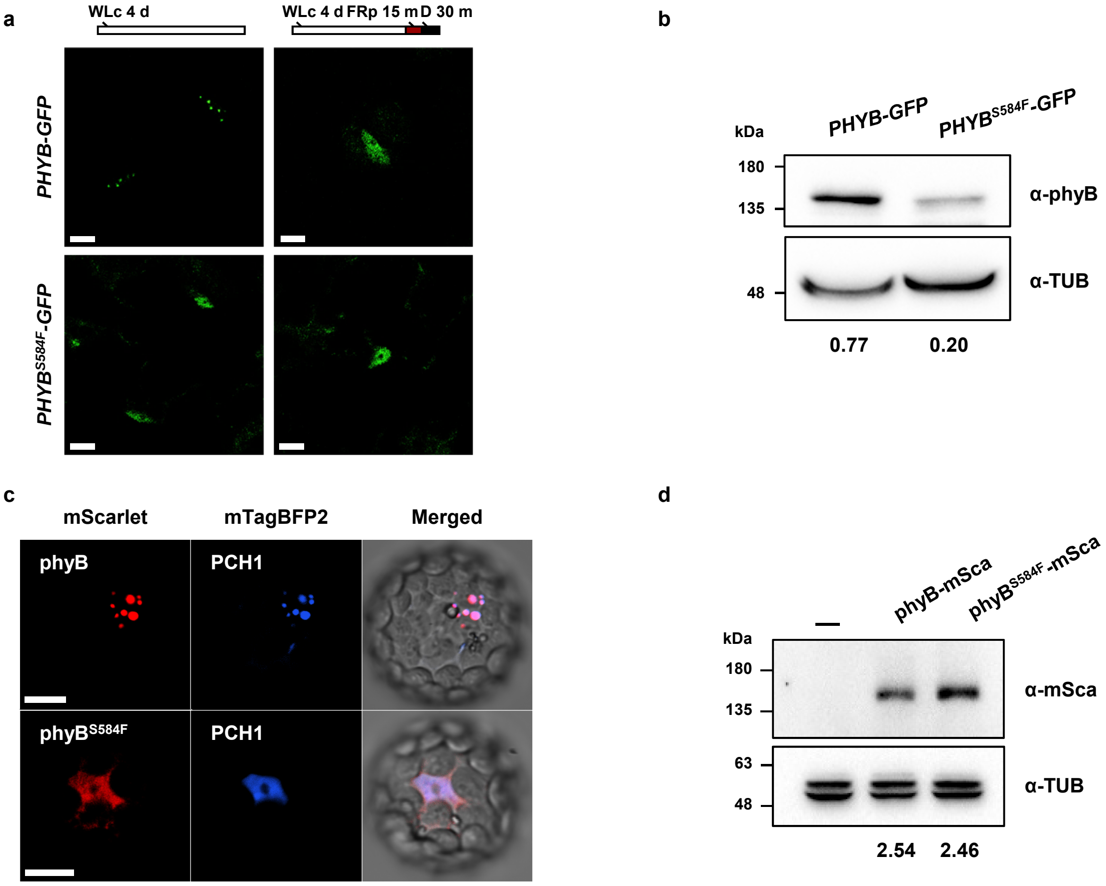

Supplementary Fig. 2 PhyB^{S584F}-GFP displays only diffuse green fluorescence nuclear signal both in transgenic plants and in protoplasts. a. Transgenic line expressing either wild-type *PHYB* or mutant *PHYB^{S584F}* were grown under white light for 4 days, and fluorescence images were taken under a confocal microscope using the GFP channel (488nm-525/25nm) either before (WLc) or after a far-red (FRp) light pulse (3 μmol/m^2/s for 15 min) was applied followed by incubation of the samples in the dark for 0.5 hours (D). Scale bars = 10 μm. **b.** PhyB protein levels in *PHYB-GFP* and *PHYB^{S584F}-GFP* transgenic lines were detected by immunoblotting using a phyB antibody (α-phyB). Tubulin was detected as a loading control using α-tubulin antibody (α-TUB). Ratios between phyB and tubulin band intensities are indicated below. **c**. mScarlet-fused phyB or phyB^{S584F} (red color) was transiently co-expressed with PCH1-mTagBFP2 (blue color) in protoplasts prepared from *phyA/phyB* double mutant. Fluorescence images were taken under a confocal microscope and merged with a white light image (Merged). Protoplast imaging experiments were performed with at least 30 protoplasts. Scale bars = 10 μm. **d.** Transiently expressed mScarlet-fused phyB or phyB^{S584F} protein levels were separated on a gradient gel and detected by immunoblotting using mScarlet antibody (α-mSca) and α-TUB. The lysate of control protoplasts is marked as “-”. Ratios between phyB and tubulin band intensities are indicated below. Source data are provided as a Source Data file.

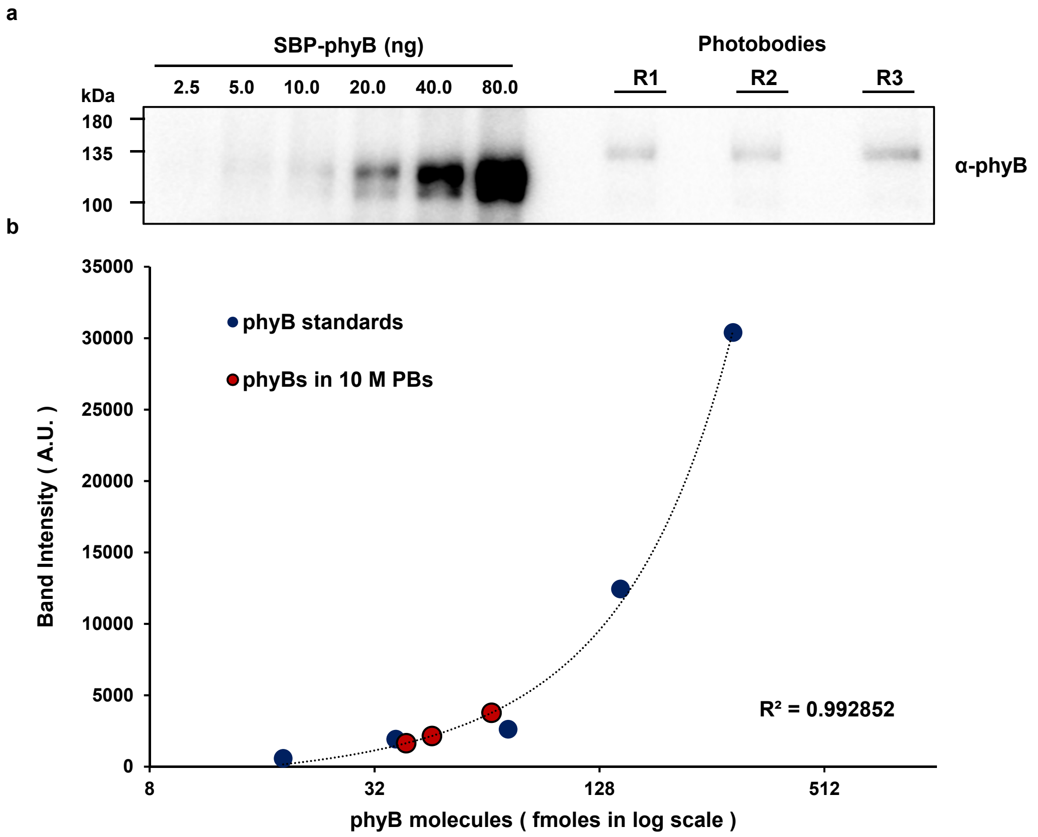

Supplementary Fig. 3 A photobody contains about 1,500 phyB dimers. a. An immunoblot assay was performed to compare the amount of phyB in 10 million (10 M) purified photobodies (Photobodies) and various amounts of recombinant phyB (SBP-phyB). Three independently sorted photobodies were used (R1, R2, R3). Both phyB-GFP and SBP-phyB were detected using a phyB antibody (α-phyB). The amounts of SBP-phyB were quantified by SDS-PAGE followed by Coomassie brilliant blue staining and comparison to BSA standards. **b**. The standard curve of phyB molecules and the estimation of phyB molecules per 10 M photobodies. The number of phyB molecules was estimated by quadratic regression (R^2 = 0.992). 80 ng of SBP-phyB was not used for the standard curve. Source data are provided as a Source Data file.

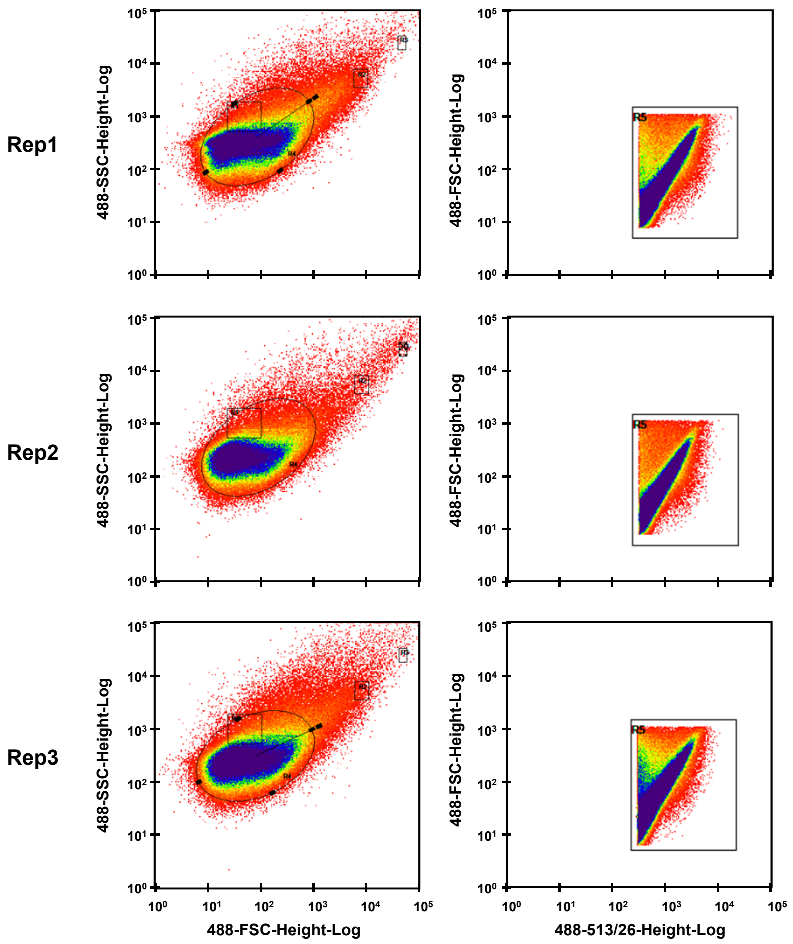

Supplementary Fig. 4 FSCxSSC and FITCxFSC plots of three sorted biological replicates. FAPS were performed with three biological replicates (Rep1 to 3). Left panels are FSCxSSC plots with scatter gates (ellipse) and right panels are FITCxFSC plots with FITC gates (rectangle). Small squares inside the FSCxSSC plots indicate the sorted positions of reference beads. All plots were captured at five minutes after the start of particle sorting.

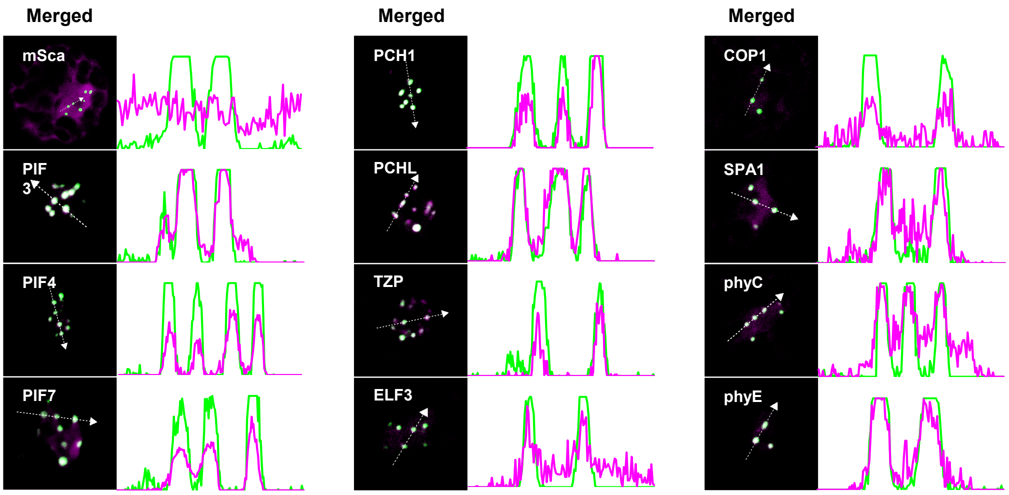

Supplementary Fig. 5 Fluorescence intensity profiles of confocal microscope images in Fig. 3. The overlap between phyB-GFP (green color) and mScarlet-fused proteins (magenta color) in the nucleus was visualized by fluorescence intensity profiles along a white dashed arrow. Green lines are the GFP fluorescence intensity profiles and magenta lines are mScarlet fluorescence intensity profiles. Source data are provided as a Source Data file.

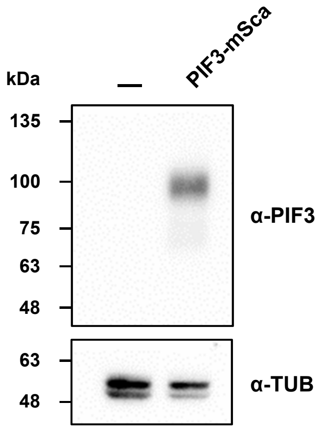

Supplementary Fig. 6 PIF3 Protein levels of endogenous and transiently expressed PIF3. PIF3-mScarlet (PIF3-mSca) was transiently expressed in protoplasts prepared from *PHYB-GFP* transgenic plants and PIF3 protein was detected using anti-PIF3 antibody (α-PIF3). Loading control were detected using anti-tubulin antibody (α-TUB). The lysate of control protoplasts is marked as "-". Transiently expressed PIF3 but not endogenous PIF3 was detected by α-PIF3, indicating the higher level of transiently expressed PIF3-mSca than that of endogenous PIF3. Source data are provided as a Source Data file.

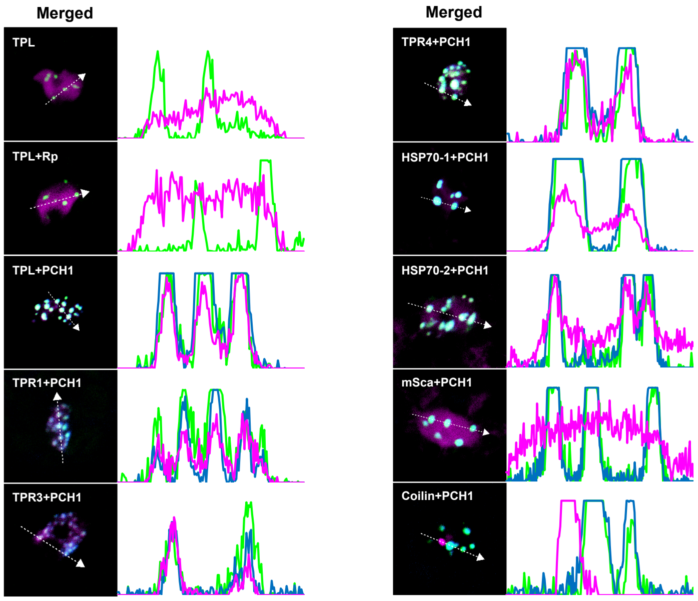

Supplementary Fig. 7 Fluorescence intensity profiles of confocal microscope images in Fig. 4. The overlap among phyB-GFP (green color), PCH1-mTagBFP2 (blue color) and mScarlet-fused proteins (magenta color) in the nucleus was visualized by fluorescence intensity profiles along a white dashed arrow. Green lines are GFP fluorescence intensity profiles, blue lines are mTagBFP2 fluorescence intensity profiles and magenta lines are mScarlet fluorescence intensity profiles. Source data are provided as a Source Data file.

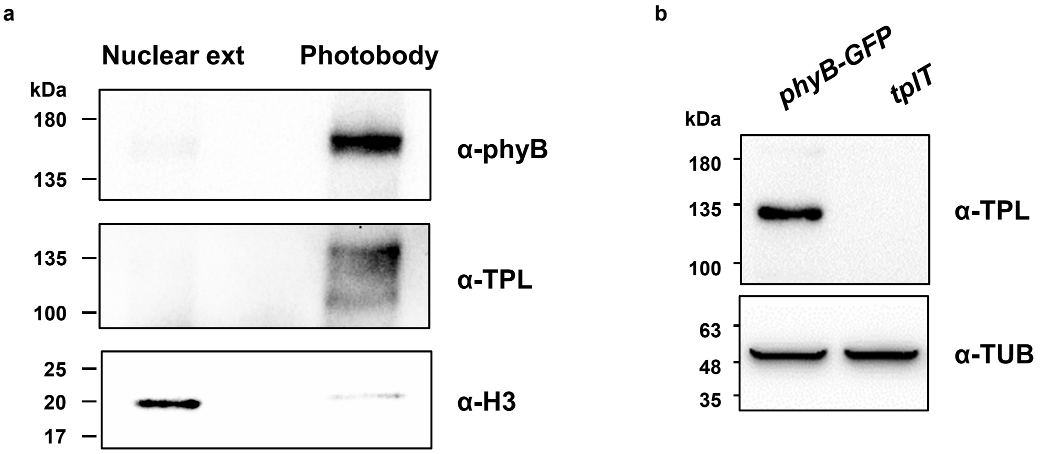

Supplementary Fig. 8 TOPLESS proteins are enriched in sorted phyB photobodies. Equal amounts (3 µg) of pre-sorted nuclear extract proteins (Nuclear ext) and sorted photobody proteins (Photobody) were immunoblotted for phyB, TOPLESS (TPL) and histone 3 using the corresponding antibodies (α-phyB, α-TPL and α-H3). The protein molecular weights of the SDS-PAGE-separated proteins are marked on the left and indicated with kDa. The protein amounts were quantified by the BCA protein assay. The right panel is control immunoblot assay showing TPL in *phyB-GFP* line but not *tplT*. Source data are provided as a Source Data file.

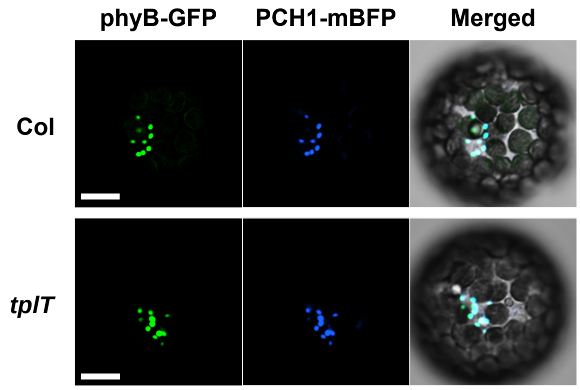

Supplementary Fig. 9 PhyB and PCH1 form photobodies in the *tpl/tpr1/tpr4* triple mutant. phyB-GFP and PCH1-mTagBFP2 (PCH1-mBFP) were transiently co-expressed in protoplasts prepared from the wild type (Col) or *tpl/tpr1/tpr4* (*tplT*) triple mutant. Fluorescence images were taken under a confocal microscope using the GFP channel (488nm-525/25nm) for phyB-GFP (green color) and the BFP channel (405nm-450/25nm) for PCH1-mBFP (blue color) and merged with a white light image (Merged). Protoplast imaging experiments were performed with at least 30 protoplasts. Scale bars = 10 μm.

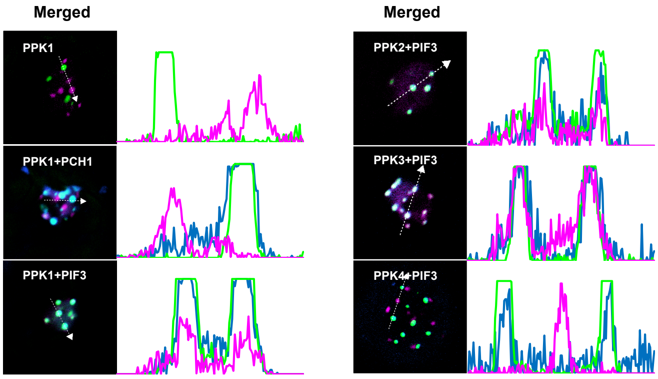

Supplementary Fig. 10 Fluorescence intensity profiles of confocal microscope images in Fig. 5. The overlap among phyB-GFP (green color), mScarlet-fused proteins (magenta color) and PCH1-mTagBFP2 or PIF3-mTagBFP2 (blue color) in the nucleus was visualized by fluorescence intensity profiles along a white dashed arrow. Green lines are GFP fluorescence intensity profiles, blue lines are mTagBFP2 fluorescence intensity profiles and magenta lines are mScarlet fluorescence intensity profiles. Source data are provided as a Source Data file.

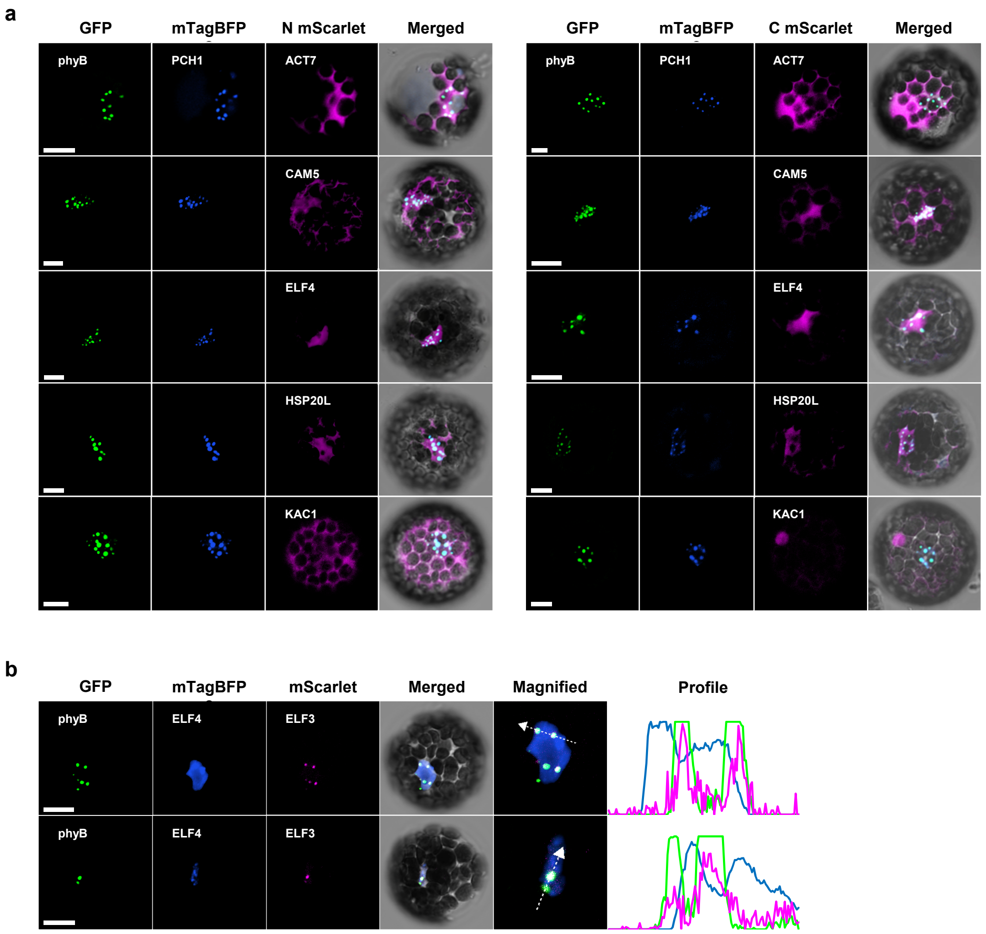

Supplementary Fig. 11 Candidate proteins that do not localize to the phyB photobody in protoplasts. a. Candidate proteins fused with mScarlet at their N-termini (N mScarlet, left panel) or C-termini (C mScarlet, right panel) were transiently co-expressed with mTagBFP2-fused PCH1 in protoplasts prepared from *PHYB-GFP* transgenic plants. The fluorescence images were taken using using the GFP channel (488nm-525/25nm) for phyB-GFP (GFP; green color), the BFP channel (405nm-450/25nm) for PCH1-mTagBFP2 (mTagBFP2; blue color), and the RFP channel (561nm-595/25nm) for mScarlet-fused proteins (mScarlet; magenta color) and merged with a white light image (Merged). Scale bars = 10 μm. **b.** ELF3 fused with mScarlet at their C-termini were transiently co-expressed with ELF4-mTagBFP2 at their C-termini in protoplasts prepared from *PHYB-GFP* transgenic plants. The fluorescence images were taken as described above. The overlap was visualized by fluorescence intensity profiles (profile) along a white dashed arrow. ELF4-mTagBFP2 displays diffuse nuclear signal in the majority of protoplasts (22 out of 30 protoplasts) (upper panel). ELF4-mTagBFP2 that infrequently forms faint nuclear condensates (8 out of 30 protoplasts) also do not overlap with phyB photobodies (lower panel). Protoplast imaging experiments were performed with at least 30 protoplasts. Scale bars = 10 μm. Source data are provided as a Source Data file.

a

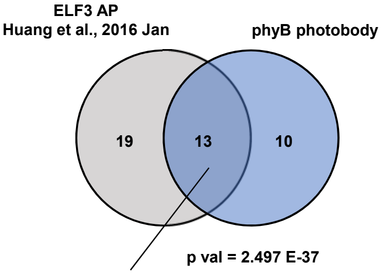

Overlapped gene AGI	Annotation
AT2G16365	PCH1
AT2G18790	phyB
AT2G25760	PPK4
AT2G25930	ELF3
AT2G32950	COP1
AT2G40080	ELF4
AT2G46340	SPA1
AT3G03940	PPK3
AT3G13670	PPK1
AT4G18130	phyE
AT5G18190	PPK2
AT5G35840	phyC
AT5G43630	TZP

b

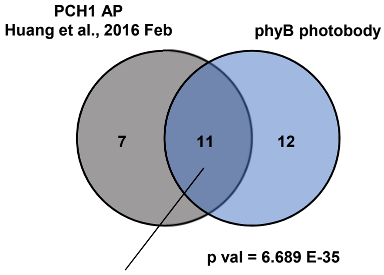

Overlapped gene AGI	Annotation
AT1G15750	TPL
AT2G16365	PCH1
AT2G18790	phyB
AT2G25930	ELF3
AT2G32950	COP1
AT2G40080	ELF4
AT2G46340	SPA1
AT3G03940	PPK3
AT4G18130	phyE
AT5G35840	phyC
AT5G43630	TZP

Supplementary Fig. 12 PhyB photobody components significantly overlap with ELF3 and PCH1 interactomes. PhyB photobody components (**Fig. 2b**) were compared with interactomes identified by the co-immunoprecipitation of ELF3 (He Huang et al., 2016 Jan) and PCH1 (He Huang et al., 2016 Feb) and visualized by Venn diagrams with p values (hypergeometric test, the number of total Arabidopsis proteins (27,416) as the population size). Tables below the Venn diagrams show identities of overlapped proteins.

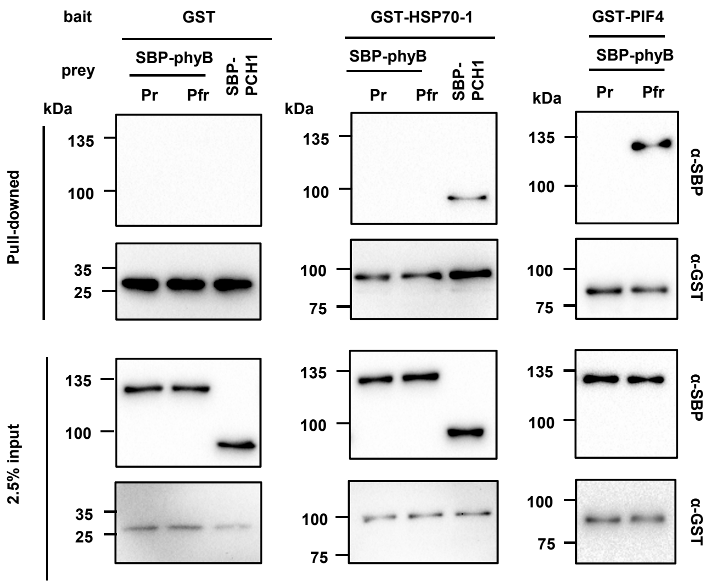

Supplementary Fig. 13 HSP70-1 interacts with PCH1 *in vitro*. *In vitro* pull-down assay showing the interaction between HSP70-1 and PCH1 but not between HSP70-1 and phyB. GST and GST-fused HSP70-1 (GST-HSP70-1) or PIF4 (GST-PIF4) were incubated with SBP-fused PCH1 (PCH1) or phyB (phyB) and pull-downed with glutathione resin. Both input (2.5% input) and pull-downed with GST proteins (pull-downed with GST) were detected with either GST antibody (α-GST) or SBP antibody (α-SBP). The Pr or the Pfr form of PhyB was obtained by irradiating a far-red light pulse (3 μmol/m^2/s, 10 min) or a red light pulse (25 μmol/m^2/s, 10 min) before the incubation and all of the following procedures were handled under safety green light. Source data are provided as a Source Data file.

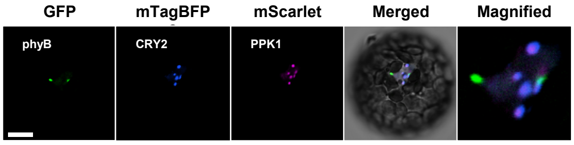

Supplementary Fig. 14 PPK1 localizes to the CRY2 nuclear body when PIF3 is not co-expressed. mScarlet-fused PPK1 was transiently co-expressed with CRY2-mTagBFP2 in protoplasts prepared from *PHYB-GFP* transgenic plants. The fluorescence images were using the GFP channel (488nm-525/25nm) for phyB-GFP (GFP; green color), the BFP channel (405nm-450/25nm) for CRY2-mTagBFP2 (mTagBFP2; blue color), and the RFP channel (561nm-595/25nm) for PPK1-mScarlet (mScarlet; magenta color) and merged with a white light image (Merged) or magnified (Magnified). Protoplast imaging experiments were performed with at least 30 protoplasts. Scale bar = 10 μm.
