# Supplementary material for: Phytochrome B photobodies are comprised of phytochrome B and its primary and secondary interacting proteins
Source: Nat Commun. 2023 Mar 27;14:1708. doi: 10.1038/s41467-023-37421-z (PMC10042835; doi:10.1038/s41467-023-37421-z)
Supplement: Supplementary file 2 — Description of Additional Supplementary Files [file 41467_2023_37421_MOESM2_ESM.pdf]

Description of Additional Supplementary Files

Supplementary Data 1 LC-MS/MS analysis of isolated photobody samples.

Supplementary Data 2 The fraction of protoplasts showing the photobody localization of a protein of interest in the presence or the absence of a co-expressed protein

Supplementary Data 3 Primers used in this study
