# Supplementary material for: Phytochrome B photobodies are comprised of phytochrome B and its primary and secondary interacting proteins
Source: Nat Commun. 2023 Mar 27;14:1708. doi: 10.1038/s41467-023-37421-z (PMC10042835; doi:10.1038/s41467-023-37421-z)
Supplement: Supplementary file 6 — Reporting Summary [file 41467_2023_37421_MOESM6_ESM.pdf]

nature portfolio

Corresponding author(s): Giltsu Choi

Last updated by author(s): Sep 9, 2022

Reporting Summary

Nature Portfolio wishes to improve the reproducibility of the work that we publish. This form provides structure for consistency and transparency in reporting. For further information on Nature Portfolio policies, see our Editorial Policies and the Editorial Policy Checklist.

Statistics

For all statistical analyses, confirm that the following items are present in the figure legend, table legend, main text, or Methods section.

n/a	Confirmed	
☐	☒	The exact sample size (*n*) for each experimental group/condition, given as a discrete number and unit of measurement
☐	☒	A statement on whether measurements were taken from distinct samples or whether the same sample was measured repeatedly
☐	☒	The statistical test(s) used AND whether they are one- or two-sided<br>*Only common tests should be described solely by name; describe more complex techniques in the Methods section.*
☒	☐	A description of all covariates tested
☒	☐	A description of any assumptions or corrections, such as tests of normality and adjustment for multiple comparisons
☐	☒	A full description of the statistical parameters including central tendency (e.g. means) or other basic estimates (e.g. regression coefficient) AND variation (e.g. standard deviation) or associated estimates of uncertainty (e.g. confidence intervals)
☐	☒	For null hypothesis testing, the test statistic (e.g. *F*, *t*, *r*) with confidence intervals, effect sizes, degrees of freedom and *P* value noted<br>*Give P values as exact values whenever suitable.*
☒	☐	For Bayesian analysis, information on the choice of priors and Markov chain Monte Carlo settings
☒	☐	For hierarchical and complex designs, identification of the appropriate level for tests and full reporting of outcomes
☒	☐	Estimates of effect sizes (e.g. Cohen's *d*, Pearson's *r*), indicating how they were calculated

Our web collection on statistics for biologists contains articles on many of the points above.

Software and code

Policy information about availability of computer code

Data collection	LC-MS/MS data were analyzed with Maxquant v1.6.10.43 and Perseus v1.5.8.
Data analysis	N/A

For manuscripts utilizing custom algorithms or software that are central to the research but not yet described in published literature, software must be made available to editors and reviewers. We strongly encourage code deposition in a community repository (e.g. GitHub). See the Nature Portfolio guidelines for submitting code & software for further information.

Data

Policy information about availability of data

All manuscripts must include a data availability statement. This statement should provide the following information, where applicable:

- Accession codes, unique identifiers, or web links for publicly available datasets
- A description of any restrictions on data availability
- For clinical datasets or third party data, please ensure that the statement adheres to our policy

Proteomics data have been deposited in PRIDE and the accession code is PXD040702.

Human research participants

Policy information about studies involving human research participants and Sex and Gender in Research.

Reporting on sex and gender	N/A
Population characteristics	N/A
Recruitment	N/A
Ethics oversight	N/A

Note that full information on the approval of the study protocol must also be provided in the manuscript.

Field-specific reporting

Please select the one below that is the best fit for your research. If you are not sure, read the appropriate sections before making your selection.

☒ Life sciences ☐ Behavioural & social sciences ☐ Ecological, evolutionary & environmental sciences

For a reference copy of the document with all sections, see nature.com/documents/nr-reporting-summary-flat.pdf

Life sciences study design

All studies must disclose on these points even when the disclosure is negative.

Sample size	To measure hypocotyl lengths, 30 seedlings were used for each sample. This number is similar to other contemporary studies in the field.
Data exclusions	No data were excluded.
Replication	We replicated experiments and representative results were shown in figures. Please refer to Statistics and Reproducibility in Method section.
Randomization	No randomization was carried out in the experimental design.
Blinding	Researchers setup and performed experiments, thus, researchers did not fully forget the experimental setup while collecting data.

Reporting for specific materials, systems and methods

We require information from authors about some types of materials, experimental systems and methods used in many studies. Here, indicate whether each material, system or method listed is relevant to your study. If you are not sure if a list item applies to your research, read the appropriate section before selecting a response.

Materials & experimental systems

n/a	Involved in the study	
☐	☒	Antibodies
☒	☐	Eukaryotic cell lines
☒	☐	Palaeontology and archaeology
☒	☐	Animals and other organisms
☒	☐	Clinical data
☒	☐	Dual use research of concern

Methods

n/a	Involved in the study	
☒	☐	ChIP-seq
☒	☐	Flow cytometry
☒	☐	MRI-based neuroimaging

Antibodies

Antibodies used	GFP polyclonal antibody_Alexa Fluor 488 (A-21311, Invitrogen) 1:100<br>H3 antibody (ab18521, Abcam) 1:1000<br>TPL antibody (AS16 3207, Agrisera) 1:1000<br>Tubulin antibody (T5168, Sigma) 1:10000<br>GST antibody (SC-138, Santa Cruz) 1:10000<br>SBP antibody (SC-101595, Santa Cruz) 1:10000<br>Goat anti-Mouse IgG HRP-conjugated (LF-SA8001, AbFrontier) 1:10000<br>Anti-Rabbit IgG HRP-conjugated (7074S, Cell Signalling) 1:10000
Validation	

GFP polyclonal antibody_Alexa Fluor 488 (A-21311, Invitrogen) Li Z, Huan C, Wang H, Liu Y, Liu X, Su X, Yu J, Zhao Z, Yu XF, Zheng B, Zhang W. TRIM21-mediated proteasomal degradation of SAMHD1 regulates its antiviral activity. EMBO Rep. 2020 Jan 7;21
H3 antibody (ab18521, Abcam) Shen JZ, Qiu Z, Wu Q, Finlay D, Garcia G, Sun D, Rantala J, Barshop W, Hope JL, Gimple RC, Sangfelt O, Bradley LM, Wohlschlegel J, Rich JN, Spruck C. FBXO44 promotes DNA replication-coupled repetitive element silencing in cancer cells. Cell. 2021 Jan 21
TPL antibody (AS16 3207, Agrisera) is validated in this study (Supplementary Figure 8b).
Tubulin antibody (T5168, Sigma) Piperno, G., et al., J. Cell Biol., 104, 289 (1987).
GST antibody (SC-138, Santa Cruz) Weng, Z., et al. 1994. Identification of Src, Fyn, and Lyn SH3-binding proteins: implications for a function of SH3 domains. Mol. Cell. Biol.14: 4509-4521.
SBP antibody (SC-101595, Santa Cruz) Stowe, I.B., et al. 2012. A shared molecular mechanism underlies the human rasopathies Legius syndrome and Neurofibromatosis-1. Genes
Dev. 26: 1421-1426.
